# From menarche to menopause: a lifespan perspective on women's health in sickle cell disease

**DOI:** 10.3389/fepid.2026.1931488

**Published:** 2026-10-16

**Authors:** Marwah Farooqui, Monica Christmas, Gelila Goba, Lewis Hsu

**Affiliations:** 1 University of Illinois Chicago, Division of Hematology & Oncology, Chicago, IL, United States; 2 University of Chicago, Department of Obstetrics and Gynecology, Chicago, IL, United States; 3 University of Miami Health System, Department of Obstetrics and Gynecology, Miami, FL, United States

**Keywords:** lifespan, menopause, reproductive health, sickle cell disease, women's health

## Abstract

Sickle cell disease (SCD) has long been associated with high childhood mortality and reduced life expectancy; however, advances in newborn screening, disease-modifying therapies, and comprehensive care have substantially improved survival. As increasing numbers of women with SCD reach midlife, new challenges are emerging that extend beyond the traditional focus on fertility and pregnancy. Despite growing recognition of reproductive health issues in SCD, menopause remains an underexplored and underrecognized phase of the disease trajectory. In this Perspective, we propose a lifespan framework for women's health in SCD that extends from menarche through menopause and healthy aging. We discuss how the underlying pathophysiology of SCD—including chronic hemolysis, inflammation, endothelial dysfunction, and progressive organ injury—intersects with reproductive transitions across the female life course. We highlight emerging evidence suggesting earlier menopause in women with SCD and examine the potential implications for pain burden, cognitive health, bone health, cardiovascular risk, iron overload, and quality of life. We further explore how symptom overlap between menopause and SCD may contribute to underdiagnosis and undertreatment during midlife. At the same time, we highlight the role of protective factors—including comprehensive multidisciplinary care, the development and evaluation of menopause-specific interventions through dedicated clinical trials in individuals with SCD, reproductive health services, and coordinated lifespan care models—in supporting healthy aging and improving long-term outcomes. Finally, we argue that menopause should be recognized as a critical component of comprehensive SCD care and research. Integrating reproductive aging into clinical practice, research agendas, and health policy represents an important opportunity to improve lifelong health outcomes, strengthen women-centered care, and advance health equity for women living with SCD worldwide.

## 1 Introduction

Sickle cell disease (SCD) is one of the most common inherited hemoglobin disorders worldwide, with the highest prevalence in sub-Saharan Africa, India, the Middle East, and the Mediterranean region. Although historically associated with high childhood mortality, survival has substantially improved in many regions of the world. This progress is driven by advances in newborn screening, preventive care, vaccination programs, hydroxyurea therapy, chronic transfusion strategies, and comprehensive sickle cell care. The global number of individuals living with SCD increased to an estimated 7.74 million in 2021, reflecting both population growth and improved survival across the lifespan (1).

**Figure 1:**
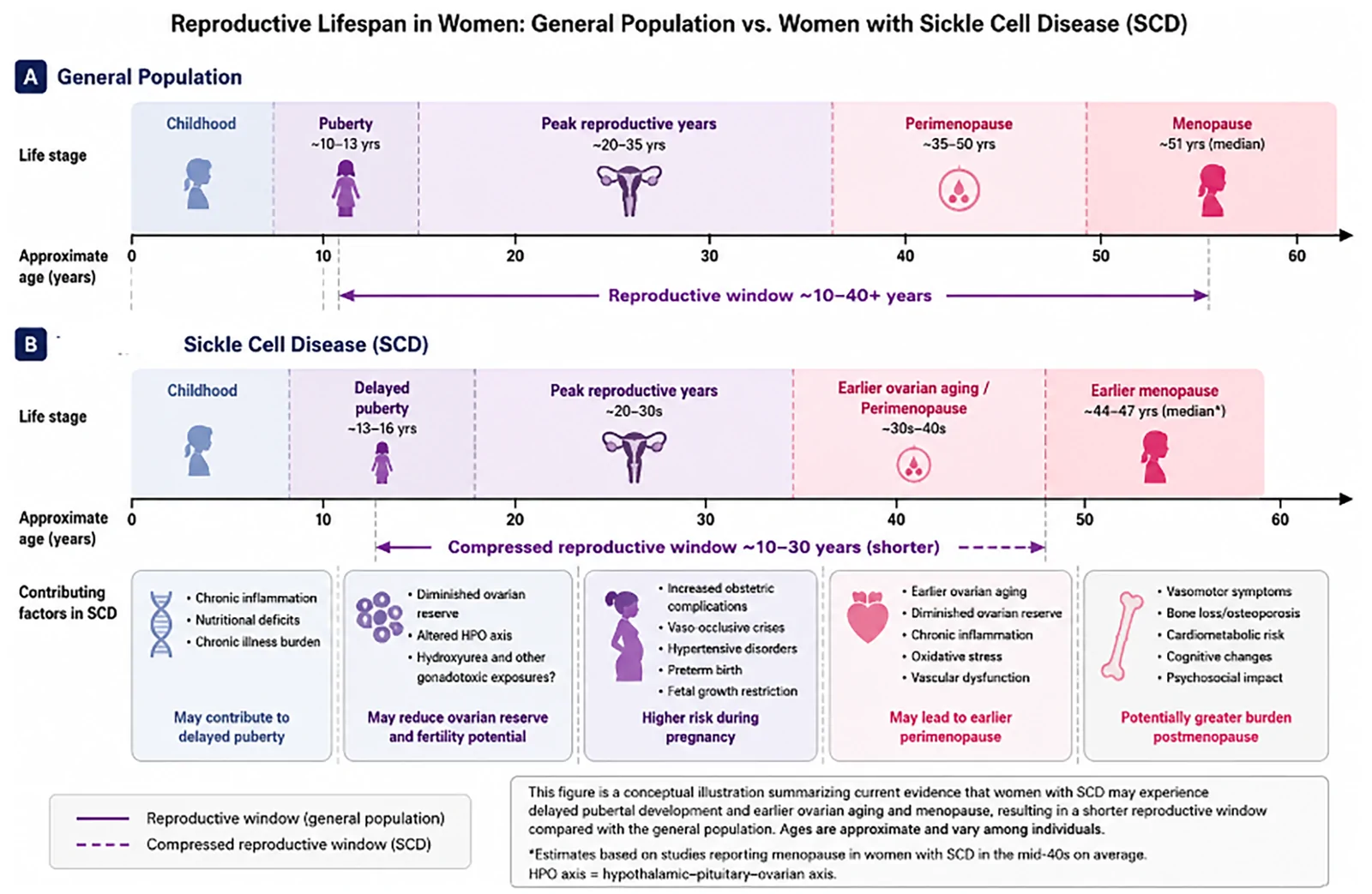
Comparison of general and sickle cell disease (SCD) fertility lifespans in women. Panel A shows general population fertility, and Panel B shows the shorter fertility lifespan of women with SCD. *Note*: This figure was generated using OpenAI's ChatGPT (GPT-4o version). The authors have fully reviewed and edited the final visual elements, confirming that all scientific labels, data relationships, and medical representations are accurate and free of automated artifact errors. Output utilized under OpenAI Terms of Service; no external copyright permissions were required.

While historical clinical literature and guidance remain predominantly focused on pediatric complications, managing care throughout the aging continuum requires a systemic transition toward a continuous, adult-focused paradigm.

In this Perspective, we propose a lifespan framework for women's health in SCD, examining how disease biology, reproductive milestones, and aging intersect across the female life course. We highlight emerging evidence regarding fertility, menopause, and age-related comorbidities, identify critical knowledge gaps, and discuss opportunities to improve women-centered care, health equity, and healthy aging for women living with SCD worldwide.

## 2 Pathophysiology of sickle cell disease across the female lifespan

SCD results from a single nucleotide substitution in the *β*-globin gene that produces hemoglobin S (HbS). Although SCD is a single gene mutation, its clinical manifestations reflect a complex interplay of red blood cell (RBC) sickling, chronic hemolysis, inflammation, endothelial dysfunction, oxidative stress, and thrombosis. These interconnected processes begin in early childhood and contribute to cumulative organ injury across the lifespan. For women, the physiological transitions of puberty, menstruation, pregnancy, and menopause interact with these underlying disease mechanisms, creating unique health challenges that evolve throughout the reproductive lifespan.

### 2.1 Core disease mechanisms

The hallmark of SCD is the polymerization of hemoglobin S (HbS) under deoxygenated conditions, leading to RBC sickling. These sickled RBCs become rigid, less deformable, and more likely to adhere to the vascular endothelium, contributing to vasoocclusion and impaired blood flow. Repeated cycles of sickling damage the RBC membrane, resulting in chronic hemolysis and a shortened RBC lifespan. Beyond vasoocclusive pain episodes, hemolysis contributes to nitric oxide depletion, oxidative stress, endothelial dysfunction, and chronic inflammation (2). It is now recognized that SCD is a complex multisystem disorder in which these overlapping processes drive progressive vascular injury and end-organ damage throughout the lifespan.

### 2.2 Organ injury and accelerated aging across the lifespan

The cumulative effects of vasoocclusion, inflammation, and vascular dysfunction begin in childhood and contribute to progressive end-organ injury throughout life. The lungs are particularly vulnerable, with individuals at risk for acute chest syndrome, pulmonary embolism, and pulmonary hypertension due to chronic hypoxia and microvascular thrombosis. The kidneys also sustain early damage; tubular dysfunction may emerge during childhood, while glomerular injury and chronic kidney disease often develop during adolescence and adulthood. Cerebrovascular injury, including silent cerebral infarction and overt stroke, contributes to cognitive impairment and reduced quality of life across the lifespan. Collectively, these processes support the concept of accelerated biological aging in SCD, whereby chronic ischemic, inflammatory, and oxidative injury may contribute to premature dysfunction across multiple organ systems (3). This framework provides a potential biological basis for considering reproductive aging alongside other manifestations of aging in SCD.

### 2.3 Genetic, environmental, and female-specific modifiers of disease severity

Although sickle cell disease is caused by a single *β*-globin gene mutation, substantial clinical heterogeneity reflects the influence of genetic modifiers, epigenetic regulation, and environmental exposures. Genetic factors such as fetal hemoglobin regulation (including variants affecting BCL11A), co-inheritance of *α*-thalassemia, APOL1-associated kidney risk variants, and pathways involved in oxidative stress and inflammation (e.g., HMOX1, Nrf2, TLR signaling) contribute to variability in disease severity, organ injury, and clinical outcomes (4). These modifiers interact with the underlying vascular pathophysiology to influence the rate of hemolysis, frequency of vasoocclusive events, and progression of end-organ damage.

Environmental and social determinants further shape disease expression across the lifespan. Exposure to air pollution, secondhand tobacco smoke, psychosocial stressors, and limited access to comprehensive care are associated with increased vasoocclusive episodes and worse overall outcomes. Importantly, female-specific physiologic states introduce additional layers of modulation. Menstruation for some individuals precipitates vasoocclusive pain episodes beginning at menarche, and hormonal fluctuations across the menstrual cycle can influence vascular tone and inflammatory balance (5). Similarly, exogenous hormonal therapies, including certain contraceptive methods, may alter thrombotic risk profiles in women with SCD. During pregnancy, physiologic changes such as increased oxygen demand, altered ventilation, and a prothrombotic state can further amplify underlying endothelial dysfunction and hemolytic stress. Together, these interactions underscore how biological sex and reproductive physiology interconnect with SCD pathophysiology to shape disease trajectories across the female lifespan.

## 3 Fertility and reproductive health: a transitional but incomplete paradigm

We propose that reproductive health in SCD has historically been framed primarily through the lens of fertility preservation and pregnancy risk, with comparatively limited attention to longitudinal reproductive aging. In clinical practice and research, menstrual health, contraceptive counseling, and fertility preservation have received increasing attention as survival improves; however, these domains are often addressed in isolation rather than as part of a continuous reproductive life-course.

Menarche marks an important inflection point in disease expression, as hormonal cycling may interact with vasoocclusive physiology, with some adolescents experiencing cyclical pain exacerbations contributing to increased healthcare utilization (5). Integrating menstrual health into routine care in SCD represents a window of opportunity for early intervention and patient-centered management.

Fertility in SCD is increasingly recognized as being influenced by a combination of disease-related and treatment-related factors. Hypoxic-ischemic injury, chronic inflammation, and potential gonadal exposure to disease-modifying therapies or transplant conditioning regimens may contribute to diminished ovarian reserve in some individuals (6).

Pregnancy represents a clinically critical period in SCD, characterized by heightened risks of vasoocclusive complications, acute chest syndrome, thromboembolism, and obstetric morbidity. Physiologic adaptations of pregnancy, including increased oxygen demand and a prothrombotic state, intersect with the underlying vasculopathy of SCD, amplifying maternal-fetal risk and necessitating multidisciplinary care (7). Women with SCD are also at increased risk for preeclampsia and medically indicated preterm delivery, contributing to adverse neonatal outcomes and significant healthcare and economic burdens associated with prematurity (8). Importantly, therapeutic decision-making during pregnancy remains complex and continuously evolving, particularly regarding disease-modifying therapies, preventative aspirin use, and analgesic strategies, reflecting persistent gaps in evidence.

We argue that while these reproductive domains are essential, they represent only part of a broader continuum. The reproductive lifespan in SCD does not end with pregnancy outcomes; rather, it transitions into midlife and menopause, which remain critically under-studied despite emerging evidence of earlier onset and significant symptom burden in this population.

### 3.1 Menopause: a new frontier in sickle cell disease

Menopause represents a critical but under-recognized phase in the care of women with SCD. The **menopausal transition (perimenopause)** is defined by the International Menopause Society as the period immediately prior to menopause (1–3 years) and the first year after menopause (9). **Menopause is defined as the permanent cessation of ovarian follicular activity. Natural menopause is recognized to occur after 12 consecutive months of amenorrhea for which there is no other obvious pathological or physiological cause.**

Available observational data suggest that menopause may occur earlier in women with SCD than in the general population. A Brazilian cohort reported menopause approximately five years earlier than expected in the general population, at approximately 46–48 years of age (10). More recent observational studies from U.S. cohorts have similarly reported mean ages of menopause in the mid-to-late 40s (11, 12). However, these studies are limited by small sample sizes, single-center designs, and retrospective data collection, which can cause significant recall bias. Larger prospective studies are needed to determine whether reproductive aging is consistently accelerated in SCD and whether the timing differs by disease genotype, severity, or treatment exposure.

Menopause is not simply a reproductive milestone but may represent a period of changing symptom burden and health risks for women with SCD. In one cohort, up to 80% of women reported a reduction in vaso-occlusive pain frequency after menopause (10), raising the possibility that hormonal changes may influence pain pathways or disease expression. However, these observations are based on limited observational data and should not be interpreted as evidence that menopause consistently improves SCD-related pain. At the same time, vasomotor symptoms, sleep disturbance, mood changes, anxiety, and fatigue may emerge or worsen during the menopausal transition (10). Because many of these symptoms overlap with manifestations of SCD, including chronic pain, fatigue, sleep disturbance, and mood symptoms, menopause-related morbidity may be underrecognized or attributed solely to SCD.

#### 3.1.1 Menopausal hormone therapy

Menopausal hormone therapy (MHT) is the most effective treatment for vasomotor symptoms and has established indications for selected women with premature or early menopause (13, 14). In the general population, the benefits and risks of MHT vary according to age, timing of initiation, formulation, route of administration, duration of therapy, and individual cardiovascular and thrombotic risk factors (15, 16). However, evidence specific to women with SCD is extremely limited. There are currently no adequately powered clinical trials evaluating the safety, efficacy, or optimal formulation and duration of MHT in women with SCD.

This distinction is particularly important when considering thrombotic risk. SCD is characterized by endothelial dysfunction, inflammation, and a prothrombotic state, and women with SCD may have additional thrombotic risk related to disease severity and other comorbidities. However, whether MHT confers an additional clinically meaningful thrombotic risk specifically in women with SCD has not been established. Concerns regarding thrombosis therefore represent an important clinical question rather than a demonstrated contraindication to MHT in this population. Future studies should examine whether thrombotic risk associated with MHT differs according to estrogen formulation, route of administration, SCD genotype, prior venous thromboembolism, disease severity, and other vascular risk factors.

Similarly, it remains unknown whether MHT modifies SCD-specific outcomes. Estrogen withdrawal has been associated with changes in vascular function in other populations (14), but whether these changes translate into clinically meaningful differences in vasoocclusive pain, endothelial function, cardiovascular events, or cerebrovascular outcomes among women with SCD is unknown. We hypothesize that the interaction between estrogen decline and the underlying vascular pathophysiology of SCD may influence disease manifestations during midlife, but this hypothesis requires prospective evaluation.

Genitourinary syndrome of menopause (GSM), characterized by vulvovaginal dryness, dyspareunia, urinary symptoms, and sexual dysfunction, is another potentially under-recognized aspect of menopause in SCD. Chronic pain, anemia-related fatigue, psychosocial stressors, and pre-existing sexual health concerns may contribute to underreporting of GSM symptoms. Low-dose vaginal estrogen is an effective treatment for GSM in the general population and has minimal systemic absorption (13). However, the prevalence of GSM and its optimal management in women with SCD have not been established. Future studies should characterize GSM burden and evaluate the safety and effectiveness of local and nonhormonal therapies in this population.

#### 3.1.2 Cognition and cerebrovascular aging

Cognitive health represents an important but understudied intersection between SCD and menopause. Cognitive complaints during menopause, including difficulties with attention, memory, and concentration, are well described in the general population and may be associated with vasomotor symptoms, sleep disruption, mood symptoms, and hormonal changes (17). Women with SCD may enter the menopausal transition with pre-existing neurovascular vulnerability related to chronic anemia, cerebral hypoxia, silent cerebral infarcts, overt stroke, and cumulative cerebrovascular injury. Consequently, their midlife hormonal transitions occur under significantly higher neurological risk than the general population.

The concept of vascular cognitive aging provides a potential framework for understanding this intersection. Chronic anemia, endothelial dysfunction, cerebral vasculopathy, and recurrent vascular injury may contribute to cumulative neurovascular damage over time. Menopausal transition is also associated with changes in vascular and metabolic health in the general population. We therefore hypothesize that reproductive aging may interact with pre-existing cerebrovascular injury in SCD and contribute to changes in cognitive function or subjective cognitive symptoms. However, there are currently insufficient data to determine whether menopause accelerates cognitive decline in women with SCD or whether MHT modifies this risk.

Future studies should incorporate both objective and subjective measures of cognition and, where feasible, neuroimaging measures of cerebrovascular injury. Longitudinal studies should examine whether cognitive trajectories across the menopausal transition differ according to prior silent cerebral infarcts, overt stroke, anemia severity, SCD genotype, and other vascular risk factors. Such studies could help distinguish menopause-related cognitive symptoms from cognitive effects attributable to cumulative SCD-related cerebrovascular injury.

#### 3.1.3 Cardiovascular, iron, bone and functional health

Menopause may also intersect with several organ systems already affected by SCD. Cardiovascular and cerebrovascular health also warrant attention as women with SCD age. Although the pattern of atherosclerotic disease in SCD may differ from that of the general population, increasing age is associated with hypertension, stroke, and other cardiometabolic complications (18). Menopause related changes in vascular and metabolic health could potentially compound these risks. We hypothesize that the menopausal transition represents an important period for cardiovascular risk assessment in women with SCD making targeted midlife screening highly clinically relevant.

Menopause has a direct impact on iron stores, and emerging observations suggest that iron indices may increase after menopause even after accounting for transfusion history (19). The cessation of menstruation eliminates a physiologic route of iron loss and may contribute to increased iron accumulation in women with SCD, which is particularly a concern among those with a history of chronic or frequent transfusions. Prospective studies are needed to determine the magnitude and clinical significance of this change and whether menopause should prompt modification of iron surveillance strategies.

Bone health is another important consideration. Estrogen decline accelerates bone resorption and contributes to loss of bone mineral density in the general population. Women with SCD may already have impaired bone health related to chronic inflammation, marrow hyperplasia, vitamin D deficiency, delayed puberty, reduced physical activity, reduced BMI, and other disease-related factors (20). Earlier menopause could therefore compound existing skeletal risk. However, the extent to which menopause contributes independently to bone loss or fracture risk in SCD has not been established. Future longitudinal studies should examine bone mineral density, fracture incidence, vitamin D status, physical activity, and other determinants of skeletal health across the menopausal transition.

Frailty and functional aging represent an additional area for investigation. The concept of accelerated biological aging in SCD is supported by cumulative vascular, inflammatory, hypoxic, and end-organ injury occurring over the life course (3). Menopause may intersect with this process through changes in muscle mass, bone health, physical activity, sleep, mood, and cardiometabolic health. We hypothesize that women with SCD may be particularly vulnerable to functional decline during midlife because menopause-related changes occur in the context of longstanding disease-related physiologic stress. However, frailty has not been adequately characterized across the menopausal transition in women with SCD. Future studies should evaluate validated measures of frailty, physical function, muscle strength, falls, multimorbidity, and quality of life alongside reproductive and disease-specific measures.

Importantly, these potential interactions may not be uniform across all women with SCD. Clinical manifestations and cumulative organ injury vary substantially by genotype, fetal hemoglobin level, co-inherited genetic modifiers, treatment exposure, and other biological and social factors (4). It is therefore plausible that the timing and consequences of menopause differ across SCD genotypes, particularly between individuals with more severe and less severe disease phenotypes. At present, however, there are insufficient data to determine whether age at menopause, menopausal symptoms, thrombotic risk, bone loss, cognitive outcomes, or frailty differ systematically by genotype. Future studies should include genotype-stratified analyses and evaluate whether disease severity and cumulative organ injury modify menopause-related outcomes.

Collectively, these considerations support the recognition of menopause as a critical phase of the SCD life course. The available evidence establishes major areas of clinical concern, justifying the integration of objective reproductive aging metrics and comprehensive risk screening into routine, longitudinal adult sickle cell care.

#### 3.1.4 Health equity and global implications

The recognition of menopause as a relevant phase of the SCD life course must be considered within the broader context of inequities that shape both SCD outcomes and women's health across the lifespan. SCD disproportionately affects populations in sub-Saharan Africa, the Middle East, India, and parts of the Americas, regions where access to comprehensive chronic disease management, reproductive health services, and menopause care remains highly variable. As a result, the intersection of SCD and menopause is not only a clinical gap but also a reflection of structural inequities in health systems and research prioritization.

Disparities in access to care extend across the reproductive lifespan. Even in resource-rich settings, access to contraception counseling, fertility preservation, high-risk obstetric care, and menopause care may be fragmented. In low- and middle-income settings, where the majority of individuals with SCD reside, structured menopause care may be unavailable within routine clinical services (14). Women with SCD may therefore enter midlife without systematic assessment of menopausal symptoms or coordinated surveillance for age-related concerns such as bone, cardiovascular, cognitive, and functional health. Fragmentation between hematology, obstetrics, gynecology, primary care, and other specialties represents an additional barrier. Menopause may fall outside the traditional scope of SCD care for hematologists, while clinicians providing menopause care may have limited experience managing the complexities of SCD. Addressing this gap will require improved communication across specialties and care pathways that recognize reproductive aging as part of longitudinal SCD management.

From a global health perspective, integrating menopause into SCD care provides an opportunity to strengthen women-centered approaches to chronic disease across the life course. However, implementation strategies must be adapted to local resources, workforce capacity, and access to both SCD and women's health services. Future efforts should therefore prioritize feasible approaches that can be incorporated into existing models of care rather than assuming that a single multidisciplinary model is applicable across all settings.

#### 3.1.5 Research gaps and future directions

Despite increasing recognition that women with SCD are reaching midlife and experiencing the menopausal transition, the evidence base remains limited. Existing studies are generally small, observational, and frequently single-center, and menopause has not been routinely incorporated as a distinct outcome in SCD cohorts or clinical trials. Consequently, fundamental questions regarding the timing of menopause, symptom burden, and its relationship to disease severity and long-term outcomes remain unanswered. Priority areas for multi-center investigative cohorts, mechanistic clinical trial endpoints, and patient-reported outcome measures are summarized comprehensively in Table 1.

**Table 1:** Current knowledge, Key Gaps, and Future Research Priorities for menopause in Women with SCD.

Domain	What is currently known	Key knowledge gap	Rationale	Recommended future studies
Timing of menopause	Small observational studies suggest menopause may occur earlier in women with SCD (10–12).	Whether earlier menopause is consistently present across populations.	Earlier menopause may have implications for bone, cardiovascular, cognitive, and overall health.	Prospective, longitudinal cohorts assessing menstrual history and age at menopause.
Menopausal transition and symptoms	Vasomotor, sleep, mood, and fatigue symptoms may overlap with manifestations of SCD.	Prevalence, severity, and trajectory of menopausal symptoms are unknown.	Symptom overlap may result in under-recognition or misattribution.	Longitudinal studies using validated menopause symptom measures and patient-reported outcomes.
Genotype and disease severity	SCD manifestations and cumulative organ injury vary substantially across genotypes and disease severity.	Whether menopause timing and menopause-related outcomes differ by genotype or disease severity is unknown.	Heterogeneity in disease biology may modify reproductive aging and midlife health.	Genotype-stratified studies evaluating menopause timing, symptoms, and long-term outcomes.
MHT	MHT is effective for menopausal symptoms in the general population, but SCD-specific data are lacking.	Safety, efficacy, optimal formulation, route, and duration in women with SCD are unknown.	SCD-specific vascular and thrombotic considerations may affect individualized treatment decisions.	Prospective clinical studies evaluating symptom outcomes and adverse events, including thrombotic outcomes.
Thrombosis	SCD is associated with endothelial dysfunction and a prothrombotic state.	Whether MHT adds clinically meaningful thrombotic risk in women with SCD is unknown.	This uncertainty may limit treatment decisions and should not be assumed to represent a contraindication.	Studies stratified by prior VTE, genotype, disease severity, estrogen formulation, and route of administration.
Pain	Limited observational data suggest some women report reduced vaso-occlusive pain after menopause (10).	The relationship between reproductive aging, hormonal changes, and pain trajectories is unclear.	Pain is a major determinant of quality of life and healthcare utilization in SCD.	Longitudinal studies examining pain frequency, severity, and analgesic use across the menopausal transition.
Cognition and cerebrovascular aging	SCD is associated with silent cerebral infarcts, stroke, and cumulative neurovascular injury.	Whether menopause modifies cognitive trajectories or interacts with pre-existing cerebrovascular injury is unknown.	Women with SCD may enter midlife with baseline neurovascular vulnerability.	Longitudinal studies incorporating objective and subjective cognitive assessments and neuroimaging where feasible.
Bone health	Women with SCD have multiple risk factors for impaired bone health; estrogen decline accelerates bone loss in the general population.	The independent contribution of menopause to bone loss and fracture risk in SCD is unknown.	Earlier menopause could compound existing skeletal vulnerability.	Longitudinal studies of bone density, fractures, vitamin D status, and physical function.
Iron burden	Cessation of menstruation eliminates a physiologic route of iron loss; women with SCD may have additional transfusion related iron exposure.	The magnitude and clinical consequences of postmenopausal changes in iron burden in SCD are unclear.	Increased iron burden may contribute to progressive organ injury.	Prospective studies of iron indices and organ iron before and after menopause.
Cardiovascular health	SCD is associated with cumulative vascular injury and age-related cardiovascular and cerebrovascular complications.	The menopause-specific contribution to cardiovascular risk in SCD is unknown.	Menopausal vascular and metabolic changes may interact with SCD-related vasculopathy.	Studies examining cardiovascular risk factors and clinical outcomes across reproductive aging.
Frailty and functional aging	SCD has been conceptualized as a condition of accelerated biological aging.	Frailty and functional trajectories across the menopausal transition have not been adequately characterized.	Menopause-related changes may occur in the setting of cumulative organ injury and physiologic stress.	Studies using validated measures of frailty, physical function, muscle strength, falls, and multimorbidity.
GSM and sexual health	GSM is common after menopause in the general population; its prevalence in women with SCD is unknown.	Prevalence, impact, and optimal treatment of GSM in SCD are not established.	Chronic pain, fatigue, and psychosocial factors may further affect sexual health and underreporting.	Studies incorporating sexual health and GSM measures and evaluating hormonal and nonhormonal therapies.
Clinical implementation	Menopause care and SCD care are frequently delivered across separate specialties.	Optimal screening, referral pathways, and models of integrated care are unknown.	Fragmented care may delay recognition and treatment.	Implementation studies evaluating feasible screening and referral strategies across care settings.
Global health and equity	Access to comprehensive SCD and menopause care varies substantially worldwide.	How to implement menopause assessment and management in resource-constrained settings is poorly understood.	The greatest burden of SCD occurs in settings with substantial healthcare resource limitations.	Equity-focused implementation research incorporating patient perspectives, local resources, and workforce capacity.

#### 3.1.6 Clinical and policy implications

Structured care pathways should implement the specific therapeutic screening protocols and care coordination guidelines outlined in Table 2, as immediate point-of-care clinical models must rely on general population consensus data. At the health-system and policy level, reproductive aging should be incorporated into future SCD clinical guidelines and continuing medical education. Funding priorities should support longitudinal studies of reproductive aging, prospective evaluation of menopause therapies, and implementation research addressing access to menopause care. Development of standardized approaches to menopause assessment within SCD care could help identify symptoms earlier and facilitate appropriate referral and treatment. These efforts should include patient perspectives and address disparities in access to both SCD and menopause care.

**Table 2:** Clinical considerations for menopause care in women With sickle cell disease.

Clinical domain	Clinical considerations	Evidence status
Recognizing the menopausal transition	Ask routinely about menstrual changes, vasomotor symptoms, sleep, mood, fatigue, genitourinary symptoms, and sexual health as women with SCD approach midlife. Consider the menopausal transition when new or changing symptoms overlap with SCD manifestations.	Established menopause-care principles; SCD-specific evidence limited.
Determining menopausal status	Document menstrual history and the timing of the final menstrual period when possible. Consider other causes of amenorrhea when the clinical picture is unclear, particularly in women with irregular menses related to SCD or other comorbidities. Consider further testing if suspect menopause transition early. In the general population, as per the International Menopause Society, hormonal testing for Anti-Müllerian hormone (AMH), Estradiol, and FSH along with antral follicle count may be used to diagnose menopause transition in women <45 (15).	General menopause principles; optimal approach in SCD requires further study.
Pain and symptom attribution	Avoid assuming that changes in pain, fatigue, sleep, mood, or other symptoms are attributable solely to SCD. Assessment should consider both SCD-related and menopause-related contributors.	Supported by symptom overlap; causal relationships remain uncertain.
Vasomotor symptoms	Assess the severity and effect of hot flashes and night sweats on sleep and quality of life. Discuss hormonal and nonhormonal treatment options using established menopause-care principles while considering individual SCD-related comorbidities and risk factors.	MHT and nonhormonal therapies are established in the general population; SCD-specific evidence is limited.
Menopausal hormone therapy	For women considering MHT, individualize treatment based on age, timing of menopause, symptoms, cardiovascular and thrombotic risk factors, prior VTE, SCD disease characteristics, formulation, route, and patient preferences. Premature or early menopause warrants particular attention to the health consequences of prolonged estrogen deficiency.	General population evidence is established; safety, efficacy, and optimal use in SCD remain uncertain.
Thrombotic risk	Recognize the underlying prothrombotic biology of SCD when evaluating MHT, but do not assume that MHT is contraindicated solely because a patient has SCD. Decisions should incorporate individual thrombotic history and risk factors.	SCD is prothrombotic; incremental thrombotic risk attributable to MHT in SCD has not been established.
Genitourinary syndrome of menopause	Ask about vaginal dryness, dyspareunia, urinary symptoms, and sexual health. Consider nonhormonal and local therapies when clinically appropriate.	Local vaginal estrogen is established for GSM in the general population; SCD-specific safety and efficacy data are lacking.
Bone health	Assess bone health in the context of existing SCD-related risk factors and menopausal estrogen decline. Consider individualized evaluation of bone mineral density and modifiable risk factors.	Bone disease is recognized in SCD and menopause; the additional contribution of menopause in SCD is uncertain.
Iron burden	Recognize cessation of menstrual blood loss as a potential change in iron balance, particularly in women with transfusion exposure. Continue appropriate iron surveillance based on individual SCD history.	Biologically plausible and supported by emerging observations; optimal menopause-specific surveillance is unknown.
Cognitive and cerebrovascular health	Consider cognitive symptoms in the context of prior silent cerebral infarcts, stroke, anemia, and other cerebrovascular complications. New cognitive concerns should not automatically be attributed solely to menopause or SCD.	SCD-related cerebrovascular injury and menopause-related cognitive symptoms are established; their interaction is not established.
Cardiovascular health	Continue age-appropriate cardiovascular risk assessment, including blood pressure and relevant metabolic risk factors, while recognizing the additional vascular burden associated with SCD.	General cardiovascular prevention principles; menopause-specific risk in SCD requires further study.
Frailty and functional health	Consider physical activity, muscle strength, falls, functional status, sleep, and multimorbidity as women with SCD age.	Accelerated aging is a proposed framework in SCD; menopause-specific effects on frailty remain understudied.
Care coordination	Incorporate reproductive aging into longitudinal SCD care and establish referral pathways to primary care, gynecology/menopause specialists, and other relevant disciplines based on individual needs.	Clinically reasonable approach; optimal models of care have not been established in SCD.
Patient-centered care	Discuss treatment options in the context of symptom burden, reproductive goals, comorbidities, treatment exposures, access to care, and patient preferences.	Established principle of individualized menopause and SCD care.

## 4 Conclusion

As women with SCD increasingly survive into midlife and older age, reproductive aging represents an important but understudied component of the SCD life course. Emerging observational data suggest that menopause may occur earlier in women with SCD, but the timing, symptom burden, and long-term health consequences of reproductive aging remain incompletely characterized. Menopause may intersect with SCD-related vascular, skeletal, neurologic, iron, cardiovascular, and functional vulnerabilities, yet the magnitude and clinical significance of many of these interactions remain uncertain.

Future research should move beyond describing age at menopause to prospectively characterize the menopausal transition and its relationship to SCD disease manifestations and aging-related outcomes. Particular priorities include understanding differences by genotype and disease severity, evaluating cognitive and cerebrovascular health, defining bone and cardiovascular consequences, and establishing the safety and efficacy of menopause therapies, including MHT, in women with SCD. Equally important will be research focused on implementation, access, and health equity.

Recognizing reproductive aging as part of comprehensive SCD care provides an opportunity to address previously overlooked aspects of women's health while generating evidence to guide individualized clinical care. A lifespan approach that integrates hematologic, reproductive, neurologic, skeletal, cardiovascular, and functional health may ultimately help ensure that improvements in SCD survival are accompanied by improvements in health and quality of life across the entire reproductive lifespan.

## Data Availability

The original contributions presented in the study are included in the article/Supplementary Material, further inquiries can be directed to the corresponding author/s.
